# Selection Decisions and Trait Preferences for Local and Imported Cattle and Sheep Breeds in Peri-/Urban Livestock Production Systems in Ouagadougou, Burkina Faso

**DOI:** 10.3390/ani9050207

**Published:** 2019-04-30

**Authors:** Regina Roessler

**Affiliations:** University of Goettingen and University of Kassel, Albrecht-Thaer-Weg 3, 37073 Goettingen, Germany; regina.roessler@agr.uni-goettingen.de; Tel.: +49-551-39-7074

**Keywords:** breeding management, breed and trait preferences, Burkina Faso, cattle, peri-/urban livestock production sector, selection decisions, sheep

## Abstract

**Simple Summary:**

Structured livestock breeding programs are needed to ensure the viability of the commercial cattle and sheep production sector in Ouagadougou, Burkina Faso. In this context, it is vitally important to better understand breeding and selection decisions of cattle and sheep breeders in the city and their trait preferences for local and imported breeds. The study interviewed 49 cattle and 31 sheep breeders to know which breeds owners keep in their herds, for which characteristics these are preferred, which criteria are used to select and replace breeding animals, and which breeding technologies are used. Results show that future breeding programs should include both local and imported cattle and sheep breeds to combine their strengths, namely the good adaptation of local breeds to prevailing production conditions, and the high production performances of imported breeds. Selection and breeding decisions for imported cattle breeds focus on higher production outputs, while the breeding management of sheep and local cattle is relatively simple. Improved breeding technologies for sheep and local cattle, simple recording schemes for the objective assessment of performance traits and planned mating schemes will help traditional cattle and sheep breeders to take conscious breeding decisions and thereby enhance animals’ production performances.

**Abstract:**

Background: Participatory approaches of designing livestock breeding programs for tropical production systems have been extensively applied for rural livestock, whereas the peri-/urban livestock production sector tends to be widely neglected. In order to ensure the viability of the commercial cattle and sheep production sector in Ouagadougou, Burkina Faso, structured breed improvement programs are needed. The study aims to better understand selection decisions of cattle and sheep breeders and their trait preferences for local and imported breeds. Methods: 49 cattle and 31 sheep breeders in peri-/urban areas of the city were approached in personal interviews. Data were analyzed in R version 3.5.1. Results: The main motivation for keeping cattle and sheep was to generate regular cash income through the selling of milk (cattle only) and surplus animals. Some (modern) breeders used imported breeds because of higher production performances. For imported cattle breeds, improved breeding technologies and management were applied to further enhance production outputs. Nevertheless, local livestock breeds were predominantly used due to their good adaptation. Conclusions: Selection decisions and trait preferences for local and imported cattle and sheep breeds were strongly based on performance traits. Especially sheep breeders, but also traditional cattle breeders, did not record performance traits and did not take conscious breeding decisions.

## 1. Introduction

Livestock keeping is a widespread activity of peri-/urban households in western sub-Saharan Africa, contributing to urban economies and people’s livelihoods [1,2]. Usually a diversity of livestock species is owned by one household, of which cattle and small ruminants are the most common [3]. Peri-/urban livestock production is mostly extensive, using small inputs of labor, commercial feed, and capital [3], being frequently integrated with crop cultivation or off-farm activities [4,5]. However, due to a steadily increasing demand for meat and milk [6], a trend towards commercialization and specialization of peri-/urban livestock production has been witnessed in sub-Saharan Africa. In Burkina Faso, intensive commercial beef cattle, sheep and pig fattening farms as well as intensive commercial dairy cattle farms have been established in or close to Ouagadougou and Bobo Dioulasso, the two largest cities of the country [5,7,8,9].

Due to this trend, livestock keepers’ breed and trait preferences as well as breeding management have changed. Accordingly, intensive sheep breeders in Ouagadougou more frequently use the Sahelian sheep breed from farther north in the country than the locally adapted Mossi sheep, because it has a higher body weight and fetches higher market prices [9]. Similarly, commercial dairy producers in the periphery of the city started upgrading the indigenous zebu breed with various European and regional breeds in order to increase the milk production output. Yet, breeding decisions are often taken individually and without following a long-term systematic crossbreeding scheme [10]. Hence, crosses are often not able to fully exploit their genetic potential [11]. 

In order to ensure the long-term viability of the commercial livestock sector in peri-/urban areas of Burkina Faso, structured livestock breeding programs are needed that combine the strengths of locally adapted and imported livestock breeds. Participatory approaches of designing livestock breeding programs for tropical production systems have been suggested that involve livestock breeders at every stage in the planning and implementation of the breeding program, including the definition of the breeding objective. They have been extensively applied for designing breeding programs for various livestock species used in rural livestock production systems, e.g., for local pigs in Vietnam [12], for llamas in Bolivia [13] and cattle in Kenya [14]. 

This study aims to better understand selection decisions of cattle and sheep breeders in peri-/urban areas of Ouagadougou, Burkina Faso, and to elucidate which breeds owners keep in their herds, for which characteristics these are preferred, and which criteria are used to select and replace male and female breeding animals. It was hypothesized that; (a) the use of breeding technologies differ between peri-/urban cattle and sheep breeders and according to livestock breed, that; (b) cattle and sheep breeders with local and imported livestock genotypes adopt different selection criteria in choosing animals for breeding, and that; (c) breeders’ trait preferences for locally adapted and imported livestock breeds are different. Results should guide the development of structured breed improvement programs for peri-/urban cattle and sheep to ensure the viability of the commercial livestock sector in Ouagadougou, and might serve as an example for other major cities in Burkina Faso and other western sub-Saharan countries.

## 2. Materials and Methods 

The study has been conducted in Ouagadougou, capital and largest city of Burkina Faso. Burkina Faso is a landlocked country in western sub-Saharan Africa. It has a dry tropical climate and three climatic zones: the Sahelian zone in the north, the north-Sudanian zone in the center and the south-Sudanian zone in the south [15]. Ouagadougou is located in the center of the country (coordinates of the city center 12°21′26″ N, 1°32′7″ W). Livestock keeping is common in the city, with 63% of households owning at least one livestock species [16].

In Ouagadougou, the UrbanFood^Plus^ project that aimed at enhancing resource use efficiency and improving food security in urban and peri-urban agriculture of West African cities was implemented from 2013–2018 [17]. Within this project, a baseline survey was realized among 181 livestock keeping households in peri-/urban areas of the city [5]. From these households, the breeders for the present study were selected, based on the following criteria: (1) considering cattle or sheep as main (most important) livestock species, (2) having at least two reproducing females of the same genetic background that calved/lambed at least once, and (3) being willing to participate in the study. The aim was to include breeders that raised local and/or imported breeds as well as crossbred animals. Additional breeders were identified by snowball sampling technique, resulting in a total of 49 cattle and 31 sheep breeders. The average number of cattle or sheep (±standard error) owned by the participating breeders was 25.7 ± 2.66 cattle and 23.8 ± 4.65 sheep, respectively (*p* > 0.05; Table 1). Of the 49 interviewed cattle breeders, 88% additionally kept poultry, 86% sheep, 80% goats, 10% pigs and 4% horses. Similarly, 84%, 80% and 13% of sheep breeders also raised goats, poultry and pigs, while 52% and 32% raised cattle or other livestock species (horses, donkey, rabbits). The owners of cattle and sheep were predominantly men (98% and 90%, respectively), with similar average age and household size between cattle and sheep breeders (*p* > 0.05 for each variable; Table 1). Significantly more cattle breeders were located in peri-urban areas (92%), while over one third of the sheep breeders were located in urban quarters of the city (*p* < 0.01; Table 1). Both cattle and sheep breeders were mostly crop-livestock farmers and had a low education level (*p* > 0.05 for each variable; Table 1). The majority of sheep breeders belonged to the Mossi ethnic group, whereas Fulani people represented more than half of the participating cattle breeders (*p* < 0.001; Table 1).

Face-to-face interviews using a semi-structured questionnaire were conducted to collect information related to the motivation of raising cattle or sheep, general breeding management, breed and trait preferences, selection criteria, as well as culling decisions and reasons. Reasons to raise cattle or sheep, reasons to prefer specific breeds, and selection and culling criteria were enquired by open-ended questions. The breeders were asked to state the selection or culling criteria, the reasons to raise cattle or sheep and the reasons for their breed preferences in order of importance which were noted verbatim and later assigned into broader categories. For breed preferences, a maximum of four reasons were stated, for all other variables a maximum of three reasons/traits were stated. Furthermore, breeders were requested to classify their breeding females with at least one parturition into one of three breed groups: (1) local breed (Fulani zebu cattle; Mossi sheep), (2) Sahelian breeds or zebu cross (Azawak and Gudali cattle and their crosses with the local Fulani zebu; pure Sahelian and Bali Bali sheep), and (3) taurine cross (crosses between the local Fulani zebu and European/international taurine cattle breeds)/crossbred sheep (crosses between the Mossi and Sahelian sheep). Breeders were then asked to select one best, one average and one poor breeding female within each breed group; in the case only two breeding females were present, the breeder was asked which female s/he considered as best and which one as poor. In total, 156 cows (100 local Fulani, 35 Sahelian, 21 European crossbred cows) and 98 ewes (61 Mossi, 12 Sahelian, 25 crossbred ewes) were selected. Next, the breeders provided a maximum of three reasons in order of importance for their ranking of each breeding female. Based on the breeders’ recall, the age, the reproduction parameters of the selected breeding females, their origin as well as the origin of their sires and dams, and the purchase price and age of breeding cows were assessed. For dairy cows, the average daily milk offtake after suckling of the calf during the last lactation was recorded. Additionally, the body weight of the selected breeding females was assessed using a weighing scale kit for cows (Agreto electronics GmbH, Raabs, Austria) or a hanging scale for ewes (Burg Wächter, Wetter, Germany). Highly agitated females and females in late gestation were excluded from the weighing.

All statistical analyses were performed using the R project, version 3.5.1 [18]. For the descriptive statistics of the data, the package pastecs was used [19]. To assess differences in the socio-economic characteristics of breeders and households, the *t*-test and the Fisher’s exact test were used for continuous variables and proportions, respectively. The proportions observed for the preferences of sire breeds and sources of dams and dams’ parents were compared using the Fisher’s exact test. For the other variables, statistical differences between groups were assessed by one-way analysis of variance. The post-hoc Tukey multiple comparison test of the R package multcomp [20] was used to confirm where differences in the herd composition occurred between breeds. The R packages emmeans [21] and multcompView [22] were used to compute least-squares means (LSM) of performance traits of the breeding females that were selected for the ranking exercise and for pairwise comparisons of LSM between breed groups and ranks within breed groups. 

For the reasons to raise cattle/sheep, to replace male and female breeding animals and to rank individual cows/ewes as best, average or poor within breed group, a weighted rank (index) was calculated. The number of times mentioned (sum of frequency) as first (i.e., most important) reason (rank 1), second important reason (rank 2) and third important reason (rank 3), were multiplied by 3 (rank 1), 2 (rank 2) or 1 (rank 3) and then added up for each reason. Finally, the sum of each reason, including zero, was divided by the total sum of all reasons to obtain the weighted rank of each reason. Q-Q plots and histograms of residuals for all traits showed a normal distribution. Therefore, linear models were used to analyze the effect of the breed group and the rank within breed group (best, average, poor) on the daily milk offtake (only cows), the body weight, the age at first parturition, the parturition interval and the number of parturitions of cows and ewes. For ewes, the effect on the twinning ability was also evaluated. No interactions were found between breed groups and rank within breed group and were hence not considered in the linear models.

For the ranking of the trait preferences for sire breeds, the number of times and the order in which the trait was mentioned by individual breeders were considered for weighting the traits (ratio-scaled evaluation) [13]. It was assumed that traits that were mentioned first were of relatively higher importance than the traits that were mentioned subsequently. Individual responses were weighted by 1 (first trait), and subsequent traits were multiplied by fractions of one, based on the total number of traits mentioned by the respective breeder. The final ranking of traits was realized based on traits evaluated on the ratio scale. Due to the low number of breeders who owned Sahelian zebu (*n* = 8) and taurine crossbred bulls (*n* = 5) or Sahelian (*n* = 4) and crossbred rams (*n* = 5), these were each combined in one group (imported sire breeds).

## 3. Results

### 3.1. Motivation for Keeping Cattle in Ouagadougou

The main motivation (weighted rank) of breeders in Ouagadougou for keeping cattle was to generate regular cash income through selling milk (0.32), surplus animals (0.17), to use cattle manure for crop fertilization (0.19), to acquire cash when needed (0.16) and to self-consume the milk (0.10). The self-consumption of meat (0.02), the use of cattle for working purposes (0.02), the sale of manure and social functions (<0.01 for each variable) were of minor importance for the interviewed cattle breeders. 

### 3.2. Breed and Trait Preferences of Cattle Breeders in Ouagadougou

The predominant cattle breed in the studied herds was the Fulani zebu (86% of all herds). Forty-three percent of the herds were only composed of this breed. Still, more than half (55%) of all cattle breeders used more than one breed, of which 37% had two, 22% three, 19% four, 7% five and 15% six breeds. Accordingly, pure Gudali and Azawak zebu cattle were present in 27% and 18% of the herds. Furthermore, 37% and 27% of the breeders had zebu or taurine crosses, respectively. The component breeds of the zebu crosses considerably varied or were unknown, making a further differentiation of this group difficult. Taurine breeds that were used for upgrading the local Fulani zebu included the Montbeliarde and Brown Swiss (each used by 18% of breeders), Holstein Friesian (16%), Tarentaise (6%), Jersey and Normande (2% each), with varying levels of exotic blood.

**Figure 1:**
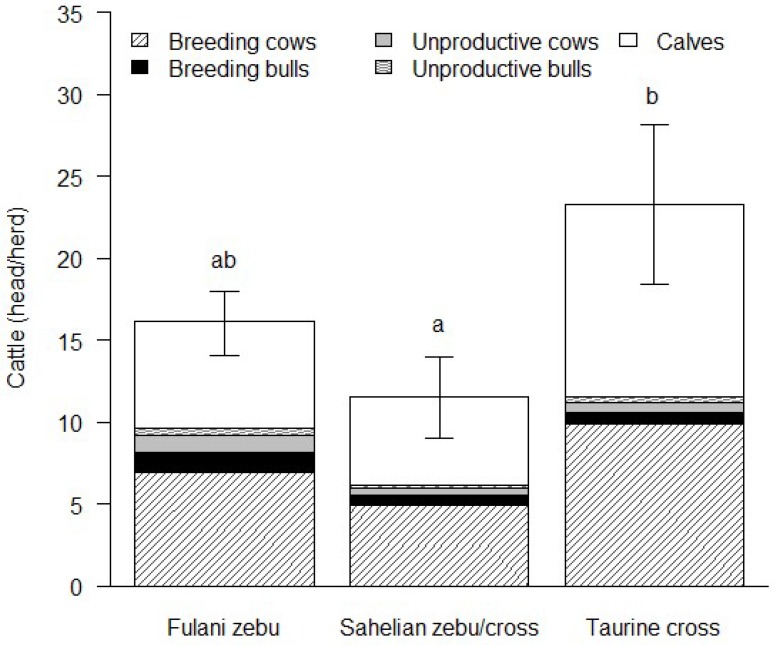
Composition and structure of cattle herds in Ouagadougou (mean ± standard error); Fulani zebu *n* = 42 herds, Sahelian zebu/cross *n* = 25 herds, taurine cross *n* = 13 herds. Means with different superscript letters are significantly different (Tukey multiple comparison of means; *p* < 0.05).

The total number of cattle kept by all cattle breeders interviewed in Ouagadougou was 1263 head, of which 53% were Fulani zebu, 3% were Gudali zebu, 9% were Azawak zebu, 11% were zebu crosses, and 24% were taurine crosses (8% each of Montbeliarde and Holstein, 6% Brown Swiss, 2% Normande and <1% each of Tarentaise, Jersey and unknown taurine crosses). The number of taurine crosses (mean ± SE) in the studied cattle herds was higher (23.5 ± 4.84 head) than of Fulani zebu (16.2 ± 1.96 head) and Sahelian zebu/crosses (11.6 ± 2.52 head) (*p* < 0.05). Calves and breeding cows constituted the major share of the studied cattle herds, while the number of bulls was low (Figure 1). Therefore, the overall bull-to-cow ratio was low (1:5), with the lowest bull-to-cow ratio (1:10) being observed in the group of the Sahelian zebu/crosses.

The comparison of observed proportions (ratio-scaled evaluation) of the reasons to prefer a specific sire breed revealed significant associations of the breed and milk yield, heritage and adaptation (all *p* < 0.001), affordability (*p* < 0.01), as well as crossbreeding/multiplication and body weight/growth (all *p* < 0.05) (Table 2). The local Fulani zebu was preferred as sire breed due to its good adaptation to the local production conditions. Also, cattle breeders considered this breed as a heritage, which implies breeders’ attachment to this breed that is passed on to the next generation. It has been used for a long time in the city region and breeders have a broad experience in keeping this breed. The Fulani zebu further requires little input (feeding, capital, health care) and is therefore affordable for the majority of cattle breeders. On the contrary, imported sire breeds (Sahelian zebu/taurine cattle breeds) were appreciated for higher milk yield and growth rate/body weight. Also, Sahelian zebu sires were preferred for crossbreeding to increase the number of crossbred cows in the herd (Table 2).

### 3.3. Breeding Management of Cattle

**Figure 2:**
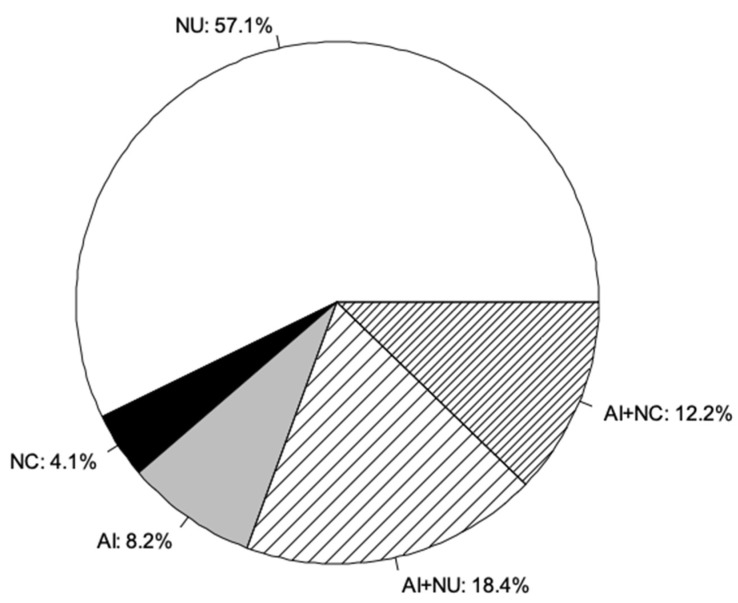
Mating methods used by cattle breeders in Ouagadougou (*n* = 49). NU: uncontrolled natural mating, NC: controlled natural mating, AI: artificial insemination.

The majority of cattle breeders used natural mating (92%), mostly uncontrolled (Figure 2). Accordingly, castration of males and separation of bulls and cows to control breeding were barely reported (8% and 14%, respectively). For natural breeding service, 82% of the cattle breeders used their own bull(s), while 22% and 11% used bulls from other breeders or communal bulls, respectively. Thirty-nine percent of breeders used artificial insemination (AI), and 8% exclusively practiced AI (Figure 2). AI services were offered by both public and private providers (53% and 47% of breeders using AI). Furthermore, estrus synchronization and hormonal stimulation of cows were applied by 20% and 12% of the cattle breeders; one breeder used embryo transfer and sexed semen, but only once. The use of reproduction technologies was limited to herds with taurine crosses and Sahelian zebu breeds. 

Selection of own bulls was realized at the age of 2.0 ± 0.20 years. On average, they were used up to the age of 5.8 ± 0.34 years. The most important reasons (weighted rank) to replace breeding bulls were fertility (0.25), inbreeding (0.18), body weight (0.16), age (0.14), and health (0.10). Breeding bulls were also replaced due to financial needs (0.08), aggressive behavior (0.03), feed scarcity (0.03), theft (0.02) and because the breeder changed to AI (0.02). 

Replacement of breeding females was usually done within the herd (Table 3). However, some differences in the source of the current female breeding stock were observed between breeds (*p* < 0.001). While the taurine crossbred cows were exclusively selected from the own herd, 21% of the (Fulani and Sahelian) zebu cows were purchased from another farm. Furthermore, 20% of the Azawak cows were provided by the Project to Support the Diffusion of the Azawak Zebu breed (PSDZA). Likewise, the dams of cows (DC) mainly originated from the own herd (81%), 15% were purchased from another farm and 3% were given by the PSDZA project. Again, other private farms were an additional source for the DC, especially for zebu cattle, while selection of DC from the own herd was comparably higher for taurine crosses (*p* < 0.01). On the contrary, the own herd was only a minor source for the sires of taurine crossbred cows which mostly originated from AI. The farmer’s own herd, private or public farms, as well as the PSDZA project, were equally important for the sourcing of sires of Sahelian zebu cows (*p* < 0.001; Table 3). 

The average selection age of female replacement stock was 1.1 ± 0.15 years. The age at acquisition of breeding cows that were sourced from other cattle herds was 3.4 ± 0.34 years, at an average purchase price of 198,500 ± 16,900 FCFA. Cows were used as long as possible, and were usually removed from the herd after 13.8 ± 0.49 years. The actual age of reproductive cows was 7.9 ± 0.23 years. The most important reasons (weighted rank) to replace cows were high age (0.31), low fertility (0.23), poor health (0.15), and low milk yield (0.13). Other, less important reasons were poor body condition of the calf (0.04), to avoid inbreeding (0.03), change of the breed, low body weight, lack of feed, and financial needs (0.02 for each variable), as well as udder and teeth problems (0.01 for each variable).

### 3.4. Animal Identification and Record Keeping of Cattle Breeders

Cattle were mostly identified by traditional identification systems, namely the coat color/pattern (43% of cattle breeders), the name (18%), the moo (10%), the appearance (4%) or brand marks (4%). Twenty-nine percent of the breeders used ear tags, 4% ear cuts, and 2% tail cutting; 10% of cattle breeders did not use any identification system. Forty-five percent of the participating cattle breeders kept animal records, of which 59%, 41% and 32% had written records about medical treatments or vaccinations, calving dates and the milk yield, respectively. Furthermore, the breed, the number of parturitions (14% each), the date of heat or insemination, the body weight, mortalities, the sex of the calf (9% each), and the weaning date (5%) were noted, yet on an irregular basis. 

#### Breeders’ Selection and Ranking Criteria for Cattle

Most important traits (ratio-scaled evaluation) that were stated for the selection of local Fulani zebu bulls were body size/growth (39%), dam’s milk yield (19%), behavioral traits (19%) and coat color/pattern (18%) (Table 4). The main criteria contributing to body size/growth mentioned verbatim by the cattle breeders were the body size and frame of the bull himself, but also of his dam and offspring, and the growth rate of the bull. Cattle breeders prioritized bulls which were docile, had a calm temperament and therefore were easy to handle. The pedigree (4%) and beautiful appearance of the bull (3%), i.e., long tail, big head, sloping hump and eye mask, were of minor importance for selection decisions for bulls of this breed. Similarly, bulls of foreign genetic background were selected based on the body size/growth (35%) and their dam’s milk yield (22%), while a beautiful appearance was of minor importance (10%). In contrast to local zebu bulls, behavior (11%) and coat color/pattern (8%) were rarely considered for selection decisions of foreign breed bulls. Besides the production traits, breed (15%) was also mentioned as selection criterion by the cattle breeders.

**Figure 3:**
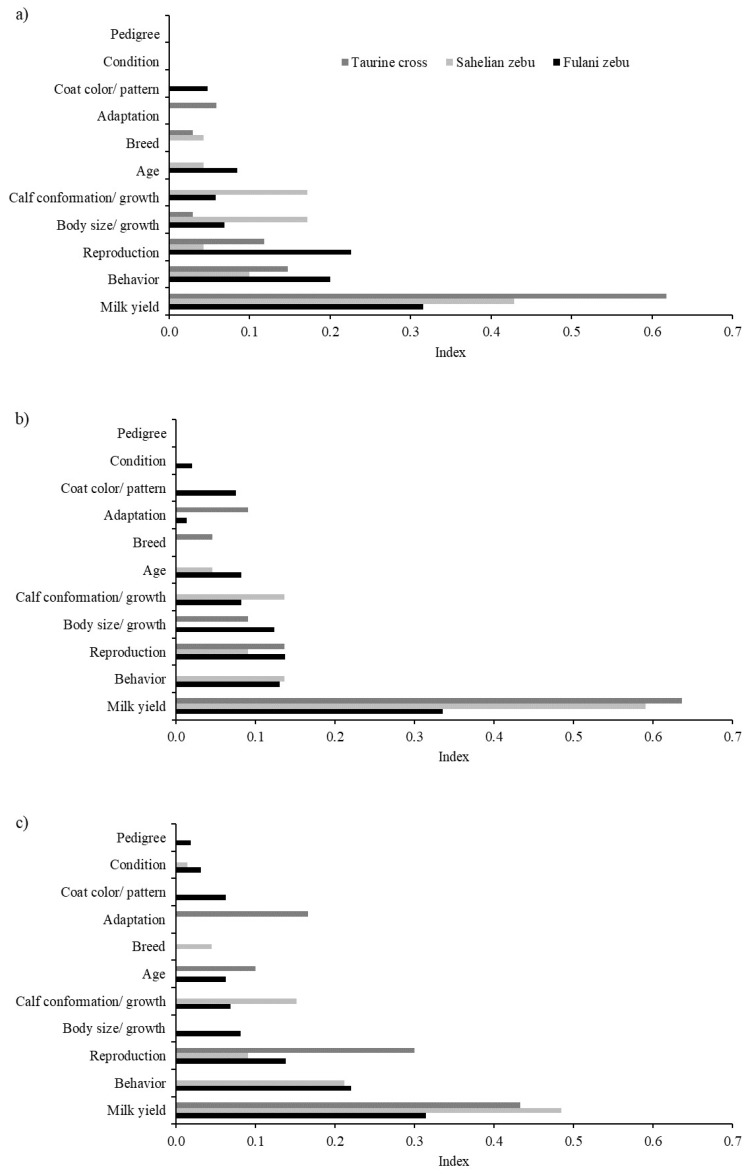
Relative importance (weighted index) of reasons for ranking individual cows as best (**a**), average (**b**) and poor (**c**) within breeds. Maximum of three reasons where the most important reason was weighted by 3, the second most important reason by 2 and the third most important reason by 1.

For the ranking of the selected cows, cattle breeders used a variety of traits (Figure 3). The most important trait was milk yield, in particular for taurine crossbred cows. Reproduction and behavioral traits were also considered for the ranking of cows, especially among the best and poorest Fulani cows, and among the poorest taurine crossbred cows. Furthermore, body size and growth performance of the cow and of her calves were of particular importance for Sahelian zebu breeds and crosses. For poor quality taurine crossbred cows, low adaptation to local production conditions was mentioned by the cattle breeders.

Cattle breeders’ ranking of cows as best, average and poor quality cows was generally confirmed by LSM of important traits, especially for production traits. Best quality cows were heavier, had a higher daily milk offtake and a higher number of parturitions than poor quality cows (*p* < 0.05). On the contrary, age at first parturition and parturition interval did not differ significantly between the best, average and poor cows (Table 5). There was also a highly significant effect of the breed on important production and reproduction traits (*p* < 0.001). Taurine crossbred cows were heavier and had a higher milk offtake than both Sahelian and Fulani zebu cows, and Sahelian zebu had a higher body weight and milk offtake than Fulani zebu cows (*p* < 0.05 for each variable). The age at first parturition and the parturition interval were lowest in taurine crossbred cows (*p* < 0.05 for each variable). Furthermore, they had a significantly higher number of parturitions than local Fulani zebu cows (*p* < 0.05) (Table 5). 

### 3.5. Motivation for Keeping Sheep in Ouagadougou

Production strategies (weighted ranks) of sheep breeders in Ouagadougou combined both economic (regular cash income through sale of animals: 0.41; cash in case of emergency: 0.33) and social functions (0.15), while the use of sheep manure as crop fertilizer was less common (0.07). Similar to cattle breeders, sheep breeders rarely stated to sell sheep manure or to self-consume sheep meat (0.02 for each variable). 

### 3.6. Breed and Trait Preferences of Sheep Breeders in Ouagadougou

More than half (52%) of the interviewed sheep breeders owned a single sheep breed, while 26% and 23% owned two or three sheep breeds, respectively. The predominant breed was the Mossi sheep (81% of the herds), followed by crossbreds between the Mossi and Sahelian sheep breeds (61% of the herds), while pure Sahelian or Bali Bali sheep were found in 16% and 13% of the herds, respectively. Three-quarter of the sheep breeders with a single breed owned the Mossi breed.

**Figure 4:**
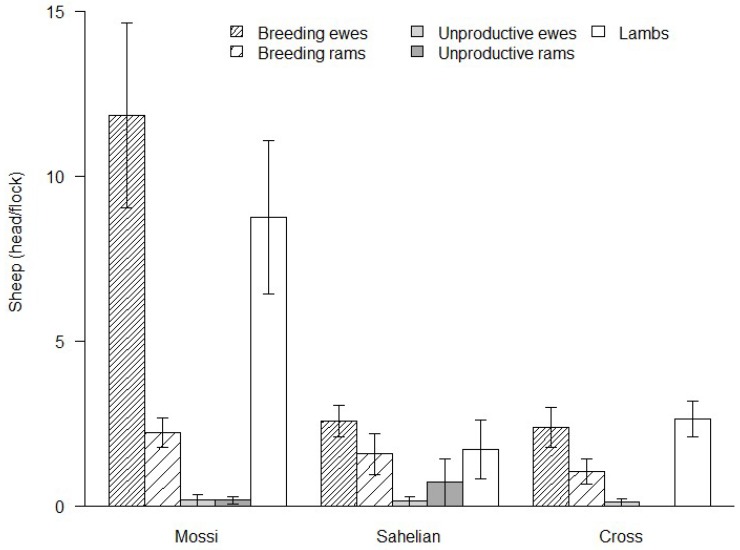
Composition and structure of sheep flocks in Ouagadougou (mean ± standard error); Mossi sheep flocks *n* = 25, Sahelian sheep flocks *n* = 9, crossbred sheep flocks *n* = 19.

The total number of sheep in the flocks that were studied amounted to 738 animals, of which 78% were Mossi, 16% were crossbreds, 3% were Sahelian and 2% were Bali Bali. Accordingly, the number of Mossi sheep in the flocks that were studied was higher (23.0 ± 5.23 head) than of Sahelian sheep (5.2 ± 1.40 head) or crossbred sheep (6.2 ± 1.38 head) (*p* < 0.05). Lambs and breeding ewes constituted the major share of flocks, in particular for the Mossi breed, whereas a relatively large proportion of rams was observed for the Sahelian breed (Figure 4). As a consequence, the ram-to-ewe ratio was higher for this breed group (1:2.8) as compared to the Mossi (1:3.3) and crossbred sheep (1:4.2) (Figure 4).

There were significant differences in the proportion of sheep breeders who chose sire breeds on the basis of good adaptation (*p* < 0.01) and large body size/good growth (*p* < 0.05) (Table 6). The Mossi breed was preferred as a sire breed due to its adaptation to the prevailing production conditions. In contrast, Sahelian/crossbred sire breeds were mostly appreciated for their greater body size/growth by the participating sheep breeders (Table 6).

### 3.7. Breeding Management of Sheep

Compared to cattle, the breeding management of sheep was simple and more homogenous. No reproduction technologies were used by the sheep breeders, and the keeping of written records for sheep was uncommon. Only one breeder occasionally recorded medical treatments of his animals. Similar to cows, the mating of ewes was mostly uncontrolled (90%). Nearly all (97%) breeders used their own breeding ram/s to mate their ewes, and uncontrolled mating by communal sires during grazing was also common (42%), while the planned mating by a ram from another farm was only mentioned by one (3%) breeder. 

Again, castration (7%) and separation of male and female sheep (10%) were barely reported by the interviewed sheep breeders. Sheep breeders identified individual animals by their coat color/pattern (58%) or their body conformation (29%), while ear cuts (7%) or tags (3%) were less common; three (10%) sheep breeders did not use any identification system. 

Usually, rams were selected for breeding at an age of 15 ± 5.6 months and replaced at 34 ± 4.1 months of age. Rams were removed from the flock due to various reasons; by far the most frequently mentioned reason (weighted rank) was financial need (0.32), followed by theft (0.16), lack of space or consumption (0.07 for each variable), as well as age, behavior, fertility problems and high feed requirements of the ram (0.06 for each variable). Least important were diseases/poor body condition (0.05), risk of inbreeding, lack of feed and small body size (0.03 for each variable), and an excess of males (0.01). 

There was no selection of ewes, because all female offspring were used for replacement. Similar to cows, ewes were used as long as possible, on average until the age of 7.6 ± 0.70 years. Consequently, the most important reason to replace ewes (weighted rank) was age (0.34), followed by fertility (0.26) and health problems (0.20). Financial need (0.06), death, poor mothering ability, poor condition of the lamb (0.04 for each variable), and high feed requirements of the ewe (0.02) were less frequently mentioned as reasons for culling. The average age of the sampled ewes was 4.4 ± 0.25 years. Also similar to cows, replacement of ewes was mostly done within the own flock, with 69% of the sampled ewes being born in the flock (Table 7). Still, sheep breeders also purchased replacement stock, and 27% and 2% of the ewes were purchased from another farm and the market, respectively. Especially for Sahelian sheep breeds, other farms were an important source for female breeding stock (58%), whereas the purchase from the market was limited to Mossi ewes (8%); however, differences between breeds were insignificant (*p* > 0.05). Interestingly, the dams of Sahelian ewes were mostly sourced from the same flock. The purchase price was similar for Mossi and crossbred ewes (Mossi: 13,000 ± 2000 FCFA, crossbred: 14,500 ± 9000 FCFA); it was considerably higher for Sahelian ewes (50,000 ± 11,000 FCFA) although these were sold at much younger age than Mossi and crossbred ewes, namely at 13 ± 2.2 months (Sahelian) compared to 33 ± 9.3 months (Mossi) and 58 ± 18.3 months (crossbred). Similar to ewes and dams of ewes, the sires of ewes were mostly selected from the breeder’s flock (77%), while 16% and 7% of ewes’ sires were communal (uncontrolled mating during free ranging) and purchased from another farm, respectively. A slight difference in the origin of sires was observed for the Sahelian sheep breeds, where they were exclusively selected from breeders’ flocks (*p* > 0.05; Table 7). 

### 3.8. Breeders’ Selection and Ranking Criteria for Sheep 

The ratio-scaled evaluation of selection criteria for rams demonstrated that Mossi and imported breed rams were mainly selected based on their body size/growth (59% and 56%, respectively) and coat color/pattern (22% and 23%, respectively). For Mossi rams, behavioral traits were also mentioned as a selection criteria by 19% of the sheep breeders. On the contrary, sheep breeders with imported breeds also based their selection decision on the breed (17%) or the presence of horns/the shape of the ears (4%) of the ram (Table 8). 

**Figure 5:**
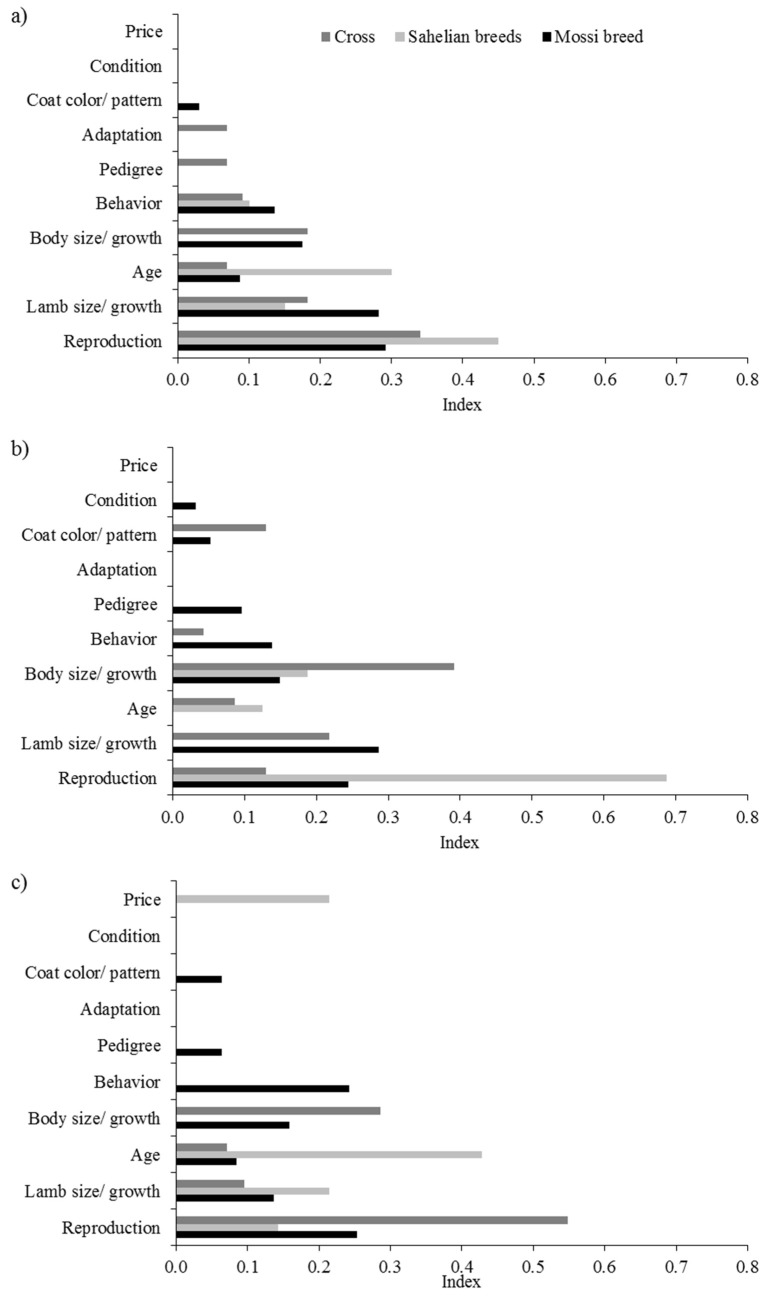
Relative importance (weighted index) of reasons for ranking individual ewes as best (**a**), average (**b**) and poor (**c**) within breeds. A maximum of three reasons, where the most important reason was weighted by 3, the second most important reason by 2 and the third most important reason by 1.

Regardless of the breed, good reproductive performance was considered as the most important trait among the best ewes (Figure 5). The reproductive performance comprised the parturition interval, the age at first parturition, the number of parturitions, the twinning ability, the number of abortions, and the sex of the lamb. The body size/growth of the lamb and the ewe herself were the second and third important reason for the Mossi and crossbred ewes, whereas the age was more important for the ranking of best Sahelian ewes. Besides the reproductive and productive performance, behavioral traits also played a role in ranking ewes as best animals, with no major differences between breeds. It is noteworthy that only crossbred ewes were appreciated for good adaptation to the prevailing production system. Also, the pedigree was only mentioned for this breed. Similar to the best ewes, the reproductive performance was considered important for ranking individual ewes as average and poor, however with considerable differences between breeds. Among poor quality ewes, the body size/growth of the ewe herself were again mentioned for the Mossi and crossbred sheep, whereas a young age tended to be more important to rank Sahelian ewes as poor. Some Mossi ewes were ranked as average and poor because of difficult behavior and their pedigree. The high price was criticized in poor Sahelian ewes, being the second most important reason mentioned for this breed (Figure 5).

There was a significant effect of breed for body weight (*p* < 0.01), with the highest body weight being determined for Sahelian and crossbred ewes, and the lowest for Mossi ewes (Table 9). Furthermore, the number of parturitions and the twinning ability of ewes differed according to the rank within breed group (*p* < 0.05 for each variable). Best quality ewes had significantly more parturitions and a higher probability of giving birth to twins than those ewes ranked poor within the same breed group (*p* < 0.05 for each variable). Other reproductive traits, namely the age at first parturition and the parturition interval, were not different between breed groups or ranks within breed group (*p* > 0.05; Table 9).

## 4. Discussion

### 4.1. Selection Decisions and Breeding Management of Local and Imported Cattle Breeds 

The primary production objective of cattle breeders in peri-/urban areas in Ouagadougou was to generate regular income through the sale of milk and surplus animals. This is in accordance with Dossa et al. [7] who showed that the main production orientation of peri-/urban cattle keepers in Bobo Dioulasso, the second largest city of Burkina Faso after Ouagadougou, was the production of milk and meat. Similar to rural cattle producers in Mali [23], cattle breeders in Ouagadougou also used sporadic animal sales to obtain emergency money, whereas the home consumption of meat and social functions were of minor importance for cattle breeders in Ouagadougou. Cattle breeders’ production objectives generally confirm the trend towards a modernization and specialization of dairy production systems close to major cities of Burkina Faso that has been reported by Roessler et al. [5]. Nevertheless, livestock keeping was commonly integrated with crop production in the present study. Hence, another important function of cattle was to provide manure to fertilize the crop fields of the breeders who mostly considered themselves as crop-livestock farmers. On the contrary, it was uncommon among the participating cattle breeders to use cattle to prepare the crop fields. Here, the production objectives of peri-/urban cattle breeders clearly differed from those of rural cattle producers in Mali [23] and Ethiopia [24], for whom the provision of draught power was an important purpose of cattle keeping.

The average herd sizes of cattle breeders in Ouagadougou ranged between 12 and 24 head, similar to 9–21 head for village herds in Niger [25]. Smaller herd sizes (2–8 head) have been reported for peri-/urban dairy cattle herds in the rift valley of Ethiopia [26], and larger herd sizes (36–40 head) for rural cattle herds in Mali [23]. The larger herd sizes of breeders who reared taurine crosses in the present study could be related to a stronger market orientation of modern dairy farms and breeders’ intention to further increase the total farm output. Due to the breeders’ production objectives and general breeding management, the studied cattle herds were largely dominated by breeding females and calves that were needed for the replacement of breeding animals. On the contrary, adult males only contributed 4–9% to the cattle herds in this study. This is in accordance with peri-urban dairy cattle herds in Niamey (Niger), to which cows and calves contributed 46–73% compared to 11–14% by males [27], whereas in village herds in southwestern Niger males represented up to 33% of the total number of cattle [25].

By and large, cattle breeders’ breed and trait preferences as well as selection criteria were in line with their primary production objectives. Especially imported sire breeds were appreciated for higher milk yield, large body size/good growth rate and higher selling price/ease of sale of calves. Concordantly, smallholder dairy producers in Jimma town (Ethiopia) preferred Holstein x indigenous zebu cows due to their high milk production [28]. Likewise, the majority of smallholder dairy producers in Niamey (Niger) preferred the Azawak breed due to its milk performance [27]. The remaining 12% of the dairy producers in Niamey appreciated the Azawak breed for its low costs [27], whereas in the present study, the affordability was only given as reason for the preference of Fulani zebu sires. Despite the above-mentioned modernization trend and promotion of imported cattle breeds, the traditional production system of settled Fulani people still dominates the peri-urban dairy production sector in Burkina Faso [29]. This is also reflected in the high number of Fulani cattle herds in the present study. The Fulani zebu breed was generally preferred due to its adaptation to local production conditions and breeders’ indigenous knowledge about this breed. Likewise, Fulani herders around Niamey gave cultural heritage and good adaptation to adverse production conditions as main reasons for keeping the local Bororo breed [27]. The Fulani zebu is well adapted to droughts and climatic hazards as well as seasonally variable feed quality and quantity that strongly affect the traditional production system of settled Fulani herders in Burkina Faso [30]. This system is further characterized by poor animal housing conditions, whereas preventive and curative medical treatments are provided to cattle [5]. Alike other indigenous livestock breeds in the region, the good adaptation of the Fulani zebu breed to the prevailing production conditions translates into lower inputs for feeding, management and health care [31], a characteristic for which the Fulani zebu cattle breed was also highly appreciated by the cattle breeders in this study.

The selection criteria for imported cattle breeds in the present study match those of smallholder dairy producers in Zimbabwe [32] and Ethiopia [28] who select cows based on milk production traits. Consistent with breeders’ reasons for their breed preferences, Sahelian zebu and taurine bulls were mainly selected based on their body size/growth performance and the dam’s milk yield. Similarly, the milk yield was the most commonly used trait for the ranking of Sahelian zebu and taurine crossbred cows, and cows which were highly ranked had a higher reported milk yield than poor quality cows. The recorded milk offtake from taurine x Fulani zebu cows over a 16-month period in peri-/urban farms of Ouagadougou was four times higher than that from purebred Fulani zebu cows [11], while the reported amounts of extracted milk from taurine crossbred cows surpassed those of Fulani cows six fold in the present study. Furthermore, taurine crossbred cows were ranked as poor because of poor reproductive performance, although the overall comparison of the age at first calving and the calving interval did not reveal a difference between the ranks of the cows. The latter could be due to the fact that cows with poorest fertility have already been removed from the herd, as this was an important reason to replace cows. The age at first parturition and the parturition interval of taurine crossbred cows as reported by cattle breeders in this study were comparable to those of crossbred cows in urban and peri-urban production systems in Ethiopia [26]. Besides fertility problems, the age, health problems and low milk yield were considered for culling of cows, like in urban production systems in Ethiopia [28]. 

The Azawak zebu is the best local milk producer in the Sahelian region [33]. According to Achard et al. [34], pure Azawak cows reared in the breeding station of Toukounous in Niger produced 1200 L per year. For Azawak cows used in modern dairy farms around Ouagadougou, the average amount of milk that was extracted for human consumption in the first six lactation months was between 2.4 and 3.9 L per day [35]. This is lower than the 4.2 L per day for the Sahelian zebu cows reported by the cattle breeders in the present study, although the amount of milk extracted from Sahelian zebu cows in modern dairy farms of Ouagadougou can be much higher [11], suggesting that Sahelian zebu herders underestimated the daily milk offtake from their cows. The ranking of best Sahelian zebu cows was also strongly based on the body size/growth of the cows and their calves. In general, cows with the highest rank had higher body weights than poor cows, and Sahelian cows were heavier than Fulani zebu cows but lighter than taurine crossbred cows. This is in accordance with Roessler et al. [11] who concluded that the body weight and growth rate of dairy cattle in peri-/urban farms in Ouagadougou were higher in imported cattle breeds than in the local Fulani cattle. 

Due to its good milk performance and adaptation to local production conditions, the Azawak zebu received special attention in national development programs in Burkina Faso. The National Union of Azawak cattle breeders (UNEAB) was established and the Project to Support the Diffusion of the Azawak Zebu breed (PSDZA) was implemented in three phases between 2002 and 2016 [36]. Also, some of the cattle breeders of the present study benefited from this project by receiving Azawak breeding stock. Still, the majority of the breeders with Sahelian zebu cattle selected their breeding cows from their own herd. Besides, the regional breeding station in Loumbila which is located close to Ouagadougou, and private farms were an important source for Sahelian zebu bulls. This breeding station was established by the Centre for the Multiplication of High-Performing Animals (CMAP) of the Ministry of Livestock Resources to support the dissemination of high-performing breeding stock through the distribution of live animals and embryos, and the provision of AI. Here, within-breed selection for the Fulani, Gudali, Azawak and Bororo zebu breeds and crossbreeding programs with Tarentaise, Montbeliarde, Holstein, Brown Swiss and Gir cattle (F1–F3 crosses), and selection programs for sheep were implemented. Also, the collection and freezing of semen from Azawak and Gudali bulls are practiced, and imported semen of taurine cattle breeds is stored at the station for the provision of AI to cattle breeders in Ouagadougou [37,38]. This public AI service was also used by the modern cattle breeders in the present study. It is particularly attractive for the cattle breeders, because it is highly subsidized by the ministry (AI service: 10,000 FCFA/cow instead of 35,000 FCFA/cow) to increase the number of artificial inseminations and the use of improved genetics [39]. Nevertheless, half of the cattle breeders also used the AI service provided by one cattle breeder who has the technical knowledge to inseminate cows with semen straws. 

A study of breeder preferences revealed that smallholder dairy producers in Tanzania were willing to keep cows with high milk yield, good fertility, easy temperament, low feed requirements and high disease resistance [40]. Similarly, cattle breeders in Ouagadougou mentioned high docility and ease to handle as reasons to rank Fulani zebu cows as best or average, and high aggressiveness as reason to rank them as poor, just after a good milk performance and fertility. In Tanzania, smallholder dairy farmers preferred an easy temperament in cows because family labor is used for feeding, milking, health management and breeding of dairy cows [40]. Also, Fulani zebu bulls were selected based on production (body size/growth, milk performance), behavior, and coat color/pattern in the present study. Likewise, the coat color and patterns were also important aesthetic reasons for the selection of dairy cows in Niger [27]. Contradictory to Belli et al. [27], adaptation was barely mentioned for the ranking of Fulani zebu cows by the cattle breeders in Ouagadougou, which could be explained by the superior adaptation of Fulani cattle as confirmed by cattle breeders’ preferences for Fulani zebu sires. Likewise, the parentage and history of progeny as well as the breed were used for selection decisions of village zebu cattle herders in Niger [25], but were of minor/no importance for selecting Fulani zebu bulls in the present study.

The higher production performances of imported cattle breeds in peri-/urban farms in Ouagadougou were a result of improved genetics, but were also associated with improved management practices and breeding technologies. The application of biotechnologies in the field of livestock breeding is often regarded as a means to improve livestock productivity in developing countries. Nevertheless, biotechnologies that are applicable for livestock breeding in developing countries are often limited because they are not adapted to the infrastructural conditions and financial resources [41]. Many biotechnologies are simply not available in developing countries, and particularly in Africa, embryo transfer and molecular genetic technologies are barely offered [42]. The use of AI is more widespread in developing countries. However, it is primarily restricted to dairy cattle in peri-urban areas, using exotic rather than local germplasm because animal identification, recording and genetic evaluation in local breeds are missing [42]. In the present study, only one breeder used embryo transfer and sexed semen once, because these techniques were poorly developed in Burkina Faso, unlike AI that was well developed for dairy cattle [37]. Appropriate training and a sufficient number of technical staff that could provide AI services were available, whereas there was a lack of sufficiently trained people for hormonal synchronization in the field [38]. The latter explains the relatively low use of hormones on modern dairy farms in Burkina Faso. 

The breeding management of the traditional Fulani cattle breeders in the present study was relatively simple. Improved reproduction technologies were not used, and castration of males and separation of bulls and cows was uncommon. With regard to the selection of breeding bulls, the majority of Fulani zebu bulls in the present study were born in the herd. In addition, breeding was largely uncontrolled and bulls and cows were used as long as possible, which might cause inbreeding problems [28]. This could explain the low production performances in Fulani zebu cows in traditional dairy farms in Ouagadougou. In contrast, rural zebu cattle breeders in Niger prevented inbreeding by selecting bulls from another herd and through castration of males [25]. Similarly, urban dairy producers in Niamey purchased their animals from local markets [27], whereas markets were completely insignificant for the acquisition of breeding stock in the present study. On the contrary, conscious selection decisions were performed by the cattle breeders with imported breeds. Less than one quarter of the sires were born in the herd, and other sources were used for the acquisition of breeding bulls; also AI was common for taurine crossbred cows. Furthermore, the replacement of bulls, and partly cows, was done to prevent inbreeding, being the second most important reason to replace bulls after fertility problems.

### 4.2. Selection Decisions and Breeding Management of Local and Imported Sheep Breeds 

According to Tindano et al. [9], sheep production in the peri-/urban area of Ouagadougou is a dual sector, with half of the producers following a traditional, extensive production style and half of them heading towards an intensification of sheep production. On the contrary, Roessler et al. [5] observed no clear trend towards an intensification of the sheep production sector unlike the dairy cattle and pig production sectors in the city. The production objectives of the sheep breeders in the present study confirm the dichotomy of the sheep production sector in Ouagadougou. On one hand, sheep were produced for regular cash income through the sale of animals. On the other hand, sheep fulfil important social functions and provide the opportunity to obtain cash in case of emergency. Similarly, Tindano et al. [9] and Dossa et al. [7] showed that sheep in Ouagadougou and Bobo Dioulasso played an important financial role, because they allowed immobilizing money until it would be spent for schooling, health care or buying of cattle. This is a clear contrast to sheep farmers in Niamey, for most of whom sheep rearing was purely a savings strategy, while the generation of income was irrelevant [43], similar to sheep farmers in Bobo Dioulasso [7]. 

The strong market orientation of sheep breeders in Ouagadougou was also reflected in the large flock sizes (23.8 sheep in Ouagadougou vs. 8.1 sheep in Bobo Dioulasso), the small share of adult males in the flocks (13% in Ouagadougou vs. 18% in Bobo Dioulasso) due to sales of male animals, and the large share of female young (< 12 months) animals (21% in Ouagadougou vs. 14% in Bobo Dioulasso) [7] to have a sufficiently large number of replacements.

Although the majority of sheep breeders in the present study were crop-livestock farmers, the use of sheep manure as crop fertilizer was uncommon. This may be due to the fact that over half of sheep breeders also owned cattle, and potentially used cattle manure as crop fertilizer. In addition, a significantly higher share of sheep breeders lived in urban areas; hence, sheep were free roaming in the city during the day, making the collection of manure or direct return to harvested crop fields through grazing more difficult.

The sheep production sector in Ouagadougou was dominated by the local Mossi sheep breed, as also reported by Tindano et al. [9]. It is small-framed, resistant to diseases, hardy and well adapted to the local environment [9,44]. Concordantly, the sheep breeders in the present study preferred Mossi rams because of their good adaptation which incurred low requirements and costs. Similar to the ranking of Fulani zebu cows, the adaptation was not considered in the ranking of Mossi ewes. This showed that the adaptation in the Mossi sheep breed was already perceived as good by the sheep breeders in Ouagadougou. In contrast to this, the low body size/poor growth performance of the local Mossi rams was disliked by the sheep breeders in the present study. Therefore, the sheep breeders’ main selection criterion used for local Mossi rams was the body size/growth. Similarly, the lambs’ and ewe’s own body size/growth were among the most frequently stated reasons for the ranking of Mossi ewes. This suggests that these traits should be genetically improved in future breeding programs for this sheep breed, even though a study involving sheep breeders in Ouagadougou revealed that the body size had no influence on breeders’ selection decisions [45]. Genetic improvement of the body size and growth rate in the Mossi sheep while maintaining its adaptation and hardiness could reduce the threat of suburban sheep breeding around Ouagadougou through indiscriminant crossbreeding with Sahelian breed rams which are appreciated for their better growth performance, but which are less resistant to diseases [44]. However, this will require an objective measurement and recording of performance traits, which was thus far not practiced by the sheep breeders in the present study, explaining the non-significant differences in the body weight of best, average and poor ewes. The appreciation of the high body size/good growth rate of Sahelian breed sheep by breeders in Ouagadougou became obvious in their stated reasons for the preferences of Sahelian rams and for the ranking of crossbred ewes. In fact, both crossbred and Sahelian ewes were heavier than the Mossi ewes in the present study. 

Irrespective of the breed, it seemed to be also important to improve the reproductive performance, especially the productive lifetime and the twinning ability, as indicated by the ranking exercise and comparison of the traits between best, average and poor ewes. Furthermore, the choice of breeding rams was based on the coat color/pattern. According to Tindano et al. [45], sheep breeders in Ouagadougou preferred white colored over bicolored breeding rams because of higher market prices for white colored animals. Similarly, sheep breeders in Niamey preferred white rams, although black sheep were more numerous in the flocks that were studied in the city [43]. 

The simple breeding management of peri-/urban sheep breeders in Ouagadougou might be an indicator of an inbreeding problem in the city’s sheep population. Firstly, the selection of ewes and rams was mostly realized from the breeders’ own flocks, while urban markets and rural farms that were relevant for sheep breeders in Bobo Dioulasso [7], were of minor/no importance to obtain breeding stock for their counterparts in Ouagadougou. Secondly, the breeding of sheep in Ouagadougou was usually uncontrolled, which concurs with findings of previous studies among sheep breeders in Ouagadougou [44] and Bobo Dioulasso [7]. Similar to sheep farmers in Bobo Dioulasso [7] and in Niamey [43], the castration of male lambs was uncommon among sheep breeders in Ouagadougou. According to Tindano et al. [44], castrated males are not allowed for the Muslim sheep sacrifice in Burkina Faso and hence fetch lower market prices. Together, all of these reasons increased the possibility that closely related animals mate. Improved reproduction technologies such as AI, which could reduce the risk of inbreeding in the city’s sheep population, were not practiced by the sheep breeders in Ouagadougou. These technologies were generally unavailable for sheep in Burkina Faso due to a lack of trained technical staff (AI) and because they have not yet been well developed for this livestock species (hormonal synchronization) [46].

## 5. Conclusions

This study confirms the present dichotomy and trend towards an intensification of the cattle and sheep production sector in Ouagadougou. This trend goes along with a change in breeders’ breed and trait preferences. Local livestock genotypes play an important role in the traditional cattle and sheep production sector, being appreciated for their good adaptation to the prevailing production conditions. However, this adaptation is also exploited by breeders with imported livestock breeds through crossbreeding of locally adapted cattle and sheep breeds with imported breeds that are preferred due to their higher production performances. Cattle and sheep breeders with both locally adapted and imported livestock breeds adopt similar selection criteria, targeted at increasing the milk yield (cows only), body size and growth performance, as well as reproductive performances of cattle and sheep. Whereas improved breeding technologies such as AI and planned mating decisions are used by cattle breeders to further enhance the milk output and body weight of imported and crossbred cows, these technologies are just not available for the sheep breeders in Ouagadougou. Furthermore, uncontrolled mating, absence of a stringent recording scheme, and the selection of replacement stock from the breeders’ own herd/flock increases the potential risk of inbreeding in local cattle and sheep breeds and therefore limit their genetic improvement. 

In developing structured breed improvement programs for peri-/urban cattle and sheep in Burkina Faso, it is important to include both, locally adapted and imported high performing livestock breeds. Selection decisions should maintain the adaptation of local livestock breeds to prevailing production conditions, while at the same time increasing the production and reproductive performances of both local and imported cattle and sheep breeds. Improved breeding technologies, planned mating and simple recording schemes for the objective assessment of selection traits are needed, especially in sheep and the local Fulani cattle breed, to further enhance their production and reproductive performances.

## Figures and Tables

**Table 1:** Socio-economic characteristics of cattle and sheep breeders in Ouagadougou.

Attribute	Cattle Breeders (*n* = 49)		Sheep Breeders (*n* = 31)		Significance Level ^2^
	Mean	SD	Mean	SD	
Age (years)	49.0	12.52	47.2	14.05	n.s.
Household size (number)	12.0	8.52	11.7	6.42	n.s.
Herd/flock size (number)	25.7	18.79	23.8	26.29	n.s.
	***n***	**%**	***n***	**%**	
Gender					n.s.
Male owner	48	98.0	28	90.3	
Female owner	1	2.0	3	9.7	
Education level					n.s.
No formal education ^1^	30	63.8	25	80.6	
Primary school	5	10.6	4	12.9	
Secondary school	9	19.1	1	3.2	
University	3	6.4	1	3.2	
Ethnicity					***
Mossi	18	36.7	25	80.6	
Fulani	28	57.1	5	16.1	
Other ethnicity	3	6.1	1	3.2	
Main occupation					n.s.
Livestock breeder	7	14.3	5	16.1	
Crop-livestock farmer	37	75.5	22	71.0	
Public employee	0	0.0	2	6.5	
Other independent job	3	6.1	2	6.5	
Retired	2	4.1	0	0.0	
Farm location					**
Urban	4	8.2	11	35.5	
Peri-urban	45	91.8	20	64.5	

^1^ Respondents with Koranic school education included. Two respondents with missing information excluded. ^2^ Significance levels: ***: *p* < 0.001, **: *p* < 0.01, *: *p* < 0.05, n.s.: not significant. *t*-test for continuous variables, Fisher’s exact test for proportions. SD: Standard deviation.

**Table 2:** Preferences for local and imported sire breeds used by cattle breeders in Ouagadougou.

Reasons for Sire Breed Preference	Fulani Zebu (Breeders *n* = 29)				Sahelian Zebu (Breeders *n* = 15)				Taurine Cattle (Breeders *n* = 13)				Significance Level ^2^
	Mentions	Ratio-Scale Evaluation ^1^		Rank	Mentions	Ratio-Scale Evaluation ^1^		Rank	Mentions	Ratio-Scale Evaluation ^1^		Rank	
	*n*	*n*	%		*n*	*n*	%		*n*	*n*	%		
Milk yield	0	0.0	0.0	-	12	7.8	33.0	1	13	12.5	78.1	1	***
Heritage/knowledge ^3^	15	12.2	29.3	2	0	0.0	0.0	-	0	0.0	0.0	-	***
Affordability	11	9.2	22.1	3	0	0.0	0.0	-	0	0.0	0.0	-	**
Adaptation	22	17.7	42.6	1	2	0.8	3.5	7	3	1.5	9.4	2	***
Crossbreeding/multiplication ^4^	0	0.0	0.0	-	3	3.0	12.8	3	0	0.0	0.0	-	*
Body size/growth	2	1.0	2.4	4	6	5.2	22.0	2	1	1.0	6.3	3	*
Selling price or selling ease of offspring	1	0.5	1.2	6	5	2.1	8.8	4	2	1.0	6.3	3	n.s.
Behavior	0	0.0	0.0	-	2	2.0	8.5	5	0	0.0	0.0	-	n.s.
Breed	0	0.0	0.0	-	2	2.0	8.5	5	0	0.0	0.0	-	n.s.
Reproduction	1	0.7	1.6	5	1	0.7	2.9	8	0	0.0	0.0	-	n.s.
Longevity	1	0.3	0.8	7	0	0.0	0.0	-	0	0.0	0.0	-	n.s.

^1^ Relative weighting of reasons on a ratio scale from 0–1, giving 1 to the first reason, and fractions of 1 to the consecutive reasons, based on the total number of reasons given by each respondent. Multiple answers were possible. ^2^ Significance levels: ***: *p* < 0.001, **: *p* < 0.01, *: *p* < 0.05, n.s.: not significant. Fisher’s exact test for comparison of observed proportions across breeds. ^3^ Heritage implies cattle breeders’ attachment to the local Fulani cattle breed that is passed on from one to the next generation. ^4^ Crossbreeding used to increase the number of crossbred cows in the herd.

**Table 3:** Source of cows ^1^ and cows’ sires and dams owned by cattle breeders in Ouagadougou (*n* = 49).

Group/Breed	Cows	Born in Herd	Private Farm	Public Farm	Communal	Project ^2^	AI
	*n*	%	%	%	%	%	%
Cows	156	75.0	20.5	0.0	n.a.	4.5	n.a.
Fulani zebu	100	75.0	25.0	0.0	n.a.	0.0	n.a.
Sahelian zebu	35	60.0	20.0	0.0	n.a.	20.0	n.a.
Taurine cross	21	100.0	0.0	0.0	n.a.	0.0	n.a.
Dams of cows	117	81.2	15.4	0.0	n.a.	3.4	n.a.
Fulani zebu	75	82.7	17.3	0.0	n.a.	0.0	n.a.
Sahelian zebu	21	66.7	14.3	0.0	n.a.	19.0	n.a.
Taurine cross	21	90.5	9.5	0.0	n.a.	0.0	n.a.
Sires of cows	117	64.1	11.1	4.3	0.9	6.0	13.7
Fulani zebu	75	86.7	12.0	0.0	1.3	0.0	0.0
Sahelian zebu	21	23.8	19.0	23.8	0.0	33.3	0.0
Taurine cross	21	23.8	0.0	0.0	0.0	0.0	76.2

^1^ Source of those cows which were used in the ranking exercise. Source of cows: *p* < 0.001, source of dams of cows: *p* < 0.01, source of sires of cows: *p* < 0.001. Fisher’s exact test. n.a.: not applicable. ^2^ Project to Support the Diffusion of the Azawak Zebu breed (PSDZA). AI: artificial insemination.

**Table 4:** Criteria used to select bulls for replacement (cattle breeders *n* = 37).

Selection Critera	Fulani Zebu (*n* = 24)				Imported Breeds (*n* = 13) ^3^			
	Mentions	Ratio-Scaled Evaluation ^1^		Rank	Mentions	Ratio-Scaled Evaluation ^1^		Rank
	*n*	*n*	%		*n*	*n*	%	
Body size/growth	18	14.5	38.7	1	10	7.0	35.0	1
Dam’s milk yield	9	7.0	18.7	2	6	4.3	21.7	2
Behavior	11	7.0	18.6	3	4	2.2	10.9	4
Coat color/pattern	10	6.7	17.8	4	2	2.5	7.5	6
Pedigree	2	1.3	3.6	5	0	0	0.0	-
Beautiful appearance ^2^	1	1.0	2.7	6	2	2.0	10.0	5
Breed	0	0	0.0	-	3	3	15.0	3

^1^ Relative weighting of reasons on a ratio scale from 0–1, for further explanation see text and footnote of Table 2. ^2^ Combines tail length, head size, hump shape and/or eye mask. ^3^ Combines Sahelian zebu and taurine cattle breeds. Multiple answers were possible.

**Table 5:** Least-square means (LSM) ± standard error (SE) for production and reproduction traits of cows ^1^ owned by cattle breeders in Ouagadougou (*n* = 49).

Effects/Levels	Cows	Body Weight	Cows	Milk Extracted	Cows	Age at First Parturition	Cows	Parturitions	Cows	Parturition Interval
	*n*	kg	*n*	L/day	*n*	Months	*n*	*n*	*n*	Months
Breed group		***		***		***		n.s.		***
Fulani zebu	99	227 ± 5.7 ^a^	100	1.8 ± 0.24 ^a^	96	50.4 ± 1.15 ^b^	100	3.1 ± 0.21 ^a^	75	20.1 ± 0.95 ^b^
Sahelian zebu	34	283 ± 9.8 ^b^	34	4.2 ± 0.41 ^b^	34	46.6 ± 1.94 ^b^	34	3.1 ± 0.37 ^ab^	27	22.6 ± 1.57 ^b^
Taurine cross	18	362 ± 13.5 ^c^	14	13.1 ± 0.63 ^c^	21	32.2 ± 2.47 ^a^	21	4.3 ± 0.47 ^b^	14	13.3 ± 2.16 ^a^
Rank within group		n.s.		***		n.s.		*		n.s.
Best	54	303 ± 8.4 ^b^	54	7.1 ± 0.36 ^b^	55	43.6 ± 1.63	56	3.9 ± 0.31 ^b^	52	19.0 ± 1.25
Average	42	294 ± 9.7 ^ab^	42	6.6 ± 0.41 ^b^	43	40.9 ± 1.87	43	3.7 ± 0.35 ^ab^	32	18.3 ± 1.55
Poor	55	276 ± 8.4 ^a^	52	5.4 ± 0.37 ^a^	53	44.6 ± 1.65	56	2.9 ± 0.31 ^a^	32	18.8 ± 1.53

^1^ Cows that were used in the ranking exercise. Significance levels: ***: *p* < 0.001, **: *p* < 0.01, *: *p* < 0.05, n.s.: not significant. LSM with different superscript letters within the same column are significantly different (*p* < 0.05).

**Table 6:** Preferences for local and imported sire breeds used by sheep breeders in Ouagadougou.

Reasons for Sire Breed Preference	Mossi (Breeders *n* = 29)				Imported Breeds ^2^ (Breeders *n* = 13)				Significance Level ^3^
	Mentions	Ratio-Scale Evaluation ^1^		Rank	Mentions	Ratio-Scale Evaluation ^1^		Rank	
	*n*	*n*	%		*n*	*n*	%		
Adaptation	16	14.1	47.5	1	1	1.0	5.4	5	**
Affordability	12	8.3	28.2	2	3	3.0	16.2	2	n.s.
Availability	3	3.0	10.2	3	1	1.0	5.4	5	n.s.
Body size/growth	3	2.7	9.1	4	9	8.5	45.9	1	*
Breed	1	1.0	3.4	5	0	0.0	0.0	-	n.s.
Knowledge	1	0.5	1.7	6	0	0.0	0.0	-	n.s.
Crossbreeding	0	0.0	0.0	-	3	2.5	13.5	3	n.s.
Selling price or selling ease of offspring	0	0.0	0.0	-	3	1.5	8.1	4	n.s.
Reproduction	0	0.0	0.0	-	1	1.0	5.4	5	n.s.

^1^ Relative weighting of reasons on a ratio scale from 0–1, giving 1 to the first reason, and fractions of 1 to the consecutive reasons, based on the total number of reasons given by each respondent. Multiple answers were possible. ^2^ Combines Sahelian breeds and crossbred sheep. ^3^ Significance levels: ***: *p* < 0.001, **: *p* < 0.01, *: *p* < 0.05, n.s.: not significant. Fisher’s exact test for comparison of observed proportions across breeds.

**Table 7:** Source of ewes ^1^ and ewes’ sires and dams owned by sheep breeders in Ouagadougou (*n* = 31).

Group/Breed	Ewes	Born in Flock	Private Farm	Market	Communal
	*n*	%	%	%	%
Ewes	98	69.4	26.5	2.0	n.a.
Mossi	61	72.1	19.7	8.2	n.a.
Cross	25	76.0	24.0	0.0	n.a.
Sahelian breeds	12	41.7	58.3	0.0	n.a.
Dams of ewes	68	77.9	22.1	0.0	n.a.
Mossi	44	81.8	18.2	0.0	n.a.
Cross	19	68.4	31.6	0.0	n.a.
Sahelian breeds	5	80.0	20.0	0.0	n.a.
Sires of ewes	68	76.5	7.4	0.0	16.2
Mossi	44	77.3	4.5	0.0	18.2
Cross	19	68.4	15.8	0.0	15.8
Sahelian breeds	5	100.0	0.0	0.0	0.0

^1^ Source of those ewes which were used in the ranking exercise. Source of ewes: *p* = 0.05 (n.s.), source of dams of ewes: *p* = 0.52 (n.s.), source of sires of ewes: *p* = 0.48 (n.s.). Fisher’s exact test. n.a.: not applicable.

**Table 8:** Criteria used to select local and imported rams for replacement (sheep breeders *n* = 26).

Selection Critera	Mossi (*n* = 17)				Imported Breeds ^3^ (*n* = 9)			
	Mentions	Ratio-Scaled Evaluation ^1^		Rank	Mentions	Ratio-Scaled Evaluation ^1^		Rank
	*n*	*n*	%		*n*	*n*	%	
Body size/growth	15	13.8	58.9	1	7	6.5	55.7	1
Coat color/pattern	8	5.2	22.0	2	4	2.7	22.9	2
Behavior	7	4.5	19.1	3	0	0.0	0.0	-
Breed	0	0.0	0.0	-	2	2.0	17.1	3
Beautiful appearance ^2^	0	0.0	0.0	-	1	0.5	4.3	4

^1^ Relative weighting of reasons on a ratio scale from 0–1, for further explanation see text and footnote of Table 2. ^2^ Combines presence of horns and shape of ears. ^3^ Combines Sahelian breeds and crossbred sheep.

**Table 9:** Least-square means (LSM) ± standard error (SE) for body weight and reproduction traits of ewes ^1^ owned by sheep breeders in Ouagadougou (*n* = 31).

Effects/Levels	Ewes	Body Weight	Ewes	Age at First Parturition	Ewes	Parturitions	Ewes	Parturition Interval	Ewes	Twinning Ability
	*n*	kg	*n*	Months	*n*	*n*	*n*	Months	*n*	%
Breed group		**		n.s.		n.s.		n.s.		n.s.
Mossi breed	56	26.9 ± 0.68 ^a^	57	20.0 ± 0.89	60	3.5 ± 0.22	49	11.6 ± 0.84	60	9.0 ± 3.29
Sahelian breeds	7	33.6 ± 1.92 ^b^	11	17.7 ± 2.02	11	4.0 ± 0.51	8	15.9 ± 2.09	11	17.2 ± 7.68
Cross	15	31.2 ± 1.34 ^b^	20	17.0 ± 1.52	25	3.0 ± 0.34	18	11.8 ± 1.41	24	13.3 ± 5.25
Rank within group		n.s.		n.s.		*		n.s.		*
Best	28	31.0 ± 1.11	32	17.7 ± 1.28	35	4.2 ± 0.31 ^b^	29	13.3 ± 1.21	35	19.8 ± 4.71 ^b^
Average	21	31.2 ± 1.28	25	18.0 ± 1.47	28	3.4 ± 0.35 ^ab^	24	13.7 ± 1.34	26	15.6 ± 5.44 ^ab^
Poor	29	29.5 ± 1.08	31	18.9 ± 1.31	35	2.9 ± 0.31 ^a^	22	12.3 ± 1.39	34	4.1 ± 4.77 ^a^

^1^ Ewes that were used in the ranking exercise. Significance levels: ***: *p* < 0.001, **: *p* < 0.01, *: *p* < 0.05, n.s.: not significant. LSM with different superscript letters within the same column are significantly different (*p* < 0.05).
